# THAP11 Functions as a Tumor Suppressor in Gastric Cancer through Regulating c-Myc Signaling Pathways

**DOI:** 10.1155/2020/7838924

**Published:** 2020-08-27

**Authors:** Jing Zhang, Huahua Zhang, Haiyan Shi, Fenghui Wang, Juan Du, Yu Wang, Yameng Wei, Wanjuan Xue, Dan Li, Yun Feng, Jing Yan, Yi Gao, Jia Li, Jiming Han

**Affiliations:** ^1^Department of Clinical Medicine, Medical College of Yan'an University, Yan'an, 716000 Shaanxi Province, China; ^2^Yan'an Key Laboratory of Chronic Disease Prevention and Research, Yan'an, 716000 Shaanxi Province, China

## Abstract

We aim to investigate the role of THAP11 (thanatos-associated protein11) in gastric cancer and its regulation mechanisms. THAP11 expression was analyzed in 51 pairs of GC tissues and the corresponding paracancerous tissues by qRT-PCR and Western blot. After THAP11 was overexpressed or knocked-down, cell proliferation, cell cycle, and apoptosis were detected in MKN-45 cells. We found that THAP11 was significantly downregulated in GC tissues and GC cell lines. Functionally, THAP11 overexpression markedly inhibited cell growth, induced G1/G0 cell-cycle arrest, and promoted cell apoptosis of MKN-45 cells, while silencing of THAP11 led to increased cell growth, increased DNA synthesis, and inhibited apoptosis. In addition, THAP11 negatively regulated the expression of c-Myc, decreased cyclinD1 protein, and increased p27 and p21 protein levels. We also found cell growth suppression induced by THAP11 was rescued by c-Myc overexpression, further confirming that THAP11 suppresses gastric cancer cell growth via the c-Myc pathway. THAP11 acts as a cell growth suppressor and exerts its role possibly through negatively regulating c-Myc pathway in gastric cancer.

## 1. Introduction

Gastric cancer (GC) is the fourth most common malignant cancer and the second leading cause of cancer-related death [1]. GC incidence in China is high, and about 150-200,000 people die of GC every year [2]. Many patients are diagnosed at advanced stages. Therefore, GC seriously threatens human health. The regulatory networks involved in GC development are multifactorial and complex, and identifying effective molecular targets is particularly important for the treatment of GC.

The THAP (Thanatos Associated Proteins) protein family is a class of evolutionarily conserved proteins with a C2CH zinc finger structure, which is known as highly conserved THAP domain. This domain has sequence-specific DNA binding ability [3]. There are totally 12 members in the THAP family, namely, THAP 0–11 [4]. Studies have reported that most THAP family members are involved in the regulation of apoptosis, angiogenesis, cell cycle progression, cell proliferation, and epigenetic gene silencing [5] and are closely related to many human diseases, such as dystonia, heart system diseases, and cancer [5–8]. Although much progress has been made in the study of THAP family members, the function of specific family member still needs to be investigated.

THAP11 is a new member of the THAP family and locates on chromosome 16q22.1. This region is heterozygously deleted in a variety of tumors. THAP11 is localized in the nucleus, and it is reported to be associated with polyglutamine disease [9]. THAP11 also functions as a tumor-promoting factor in teratocarcinoma of mice [10]. Moreover, THAP11 is widely expressed in normal tissues such as kidney, heart, liver, brain, lung, and pancreas with particularly high expression in cardiac tissues. Interestingly, it is upregulated in lung cancer and ovary cancer [11, 12] and downregulated in liver cancer, kidney cancer, and colon cancer [8, 12, 13], suggesting the important role of THAP11 in cancer. At present, there is no report on the role of THAP11 in GC.

In the present study, we analyzed the expression pattern and function of THAP11 in tissues of GC patients and GC cell lines, and found that THAP11 inhibited the proliferation of GC cells though regulating c-Myc expression.

## 2. Materials and Methods

### 2.1. Human GC Tissue Specimens and Cell Lines

A total of 51 cases of GC tissues and its paracancerous tissues were obtained from patients who were treated in the Department of Oncology Surgery, The First Affiliated Hospital of Medical College, Yan'an University, from April 2016 to August 2018. They were confirmed by pathology and had complete clinical and pathological data. All cases were not treated with radiotherapy or chemotherapy before surgery. Of the 51 patients, 29 were males and 22 were females. They were aged 26-75 years, and the mean age was 54 years. The study was approved by the Ethical Committee of Yan'an University. Informed consent was obtained from each patient.

The human gastric mucosal cell line GES-1 and human GC cell lines including SGC-7901, BGC-823, and MKN-45 were kindly provided by Prof. Huang Chen (Key Laboratory of Environment and Gene Related to Diseases, Xi'an Jiaotong University Health Science Center, China). For culture of SGC-7901 and BGC-823 cells, RPMI-1640 medium (Gibco BRL, NY, USA) supplemented with 10% fetal bovine serum (Gibco) was used, and for culture of MKN-45 and GES-1 cells, DMEM medium (Gibco) supplemented with 10% fetal bovine serum (Gibco) was used. All the cells were cultured at 37°C with 5% CO_2_.

### 2.2. Transient Transfection

THAP11 and c-Myc overexpression plasmids, pGV141-THAP11 and pGV141- c-Myc, and the negative control plasmids, pGV141, were purchased from GENECHEM (Shanghai, China). Small interfering RNA (siRNA) targeting THAP11 (si-THAP11) and control siRNA were from Gene-Pharma (Shanghai, China). The siRNA sequences were shown in Table 1. MKN-45 cells were cultured until the cell confluence reached 60-80%, and the corresponding overexpression plasmids or RNA interference fragments was transfected with lipofectamine 2000 (Thermo Fisher Scientific) according to the manufacturer's instructions.

### 2.3. Quantitative Real-Time Polymerase Chain Reaction (qRT-PCR)

Total RNAs were extracted from GC tissues and GC cell lines using Trizol Reagent (Invitrogen, Carlsbad, CA, USA). The RNA concentration was detected by Nanodrop (Thermo Fisher Scientific Inc., DE, USA). Reverse transcription of RNA into cDNA was performed with the PrimeScript RT kit (Takara, Dalian, China). THAP11 and c-Myc mRNA were measured with qRT-PCR using SYBR Green PCR kit (Takara). The primer sequences were listed in Table 2. GAPDH was used as internal control. The real-time quantitative PCR reaction conditions were set according to the IQ5 Multicolor qRT-PCR Detection System (Bio-Rad, Hercules, CA, USA). The 2^−ΔΔCt^ method was used to calculate the relative mRNA level.

### 2.4. Western Blot

Human GC tissues, GC cells, and GES-1 cells were lysed with RIPA buffer (Cell Signaling Technology, Boston, MA) containing the protease inhibitor (Roche, Indianapolis, IN, USA) to extract the total protein. Each group of cells was subjected to protein quantification using a BCA quantification kit (Pierce). A 10% SDS-PAEG gel was prepared to separate protein, and the protein was transferred to a PVDF membrane. After blocking, the membrane was incubated with primary antibodies at 4°C overnight. After washing, the corresponding secondary antibodies were added and incubated for 1.5 h at room temperature. The antibodies used were listed in Table 3. Color development was performed with ECL. The image was detected and acquired by SyngeneGBox (Syngene, Cambridge, UK), and the luminescence signal was recorded. The loading control was GAPDH.

### 2.5. MTT Assay

MKN-45 cells were transfected as above described. A total of 5000 cells were seeded into each well of 96-well plates. A total of 10 *μ*l MTT (Sigma, St. Louis, MO, USA) was added to each well at 24 h, 48 h, and 72 h after transfection and incubated for 4 h at 37°C. After dissolving the precipitate in 150 *μ*l DMSO, the absorbance at 490 nm was measured with POLARstar OPTIMA microplate (BMG Labtechnologies, Germany).

### 2.6. Cell Counting

MKN cells were collected at 24, 48, and 72 h after transfection, and cell number of each group was counted with haemocytometer.

### 2.7. Cell Cycle Analysis

At 24 h after transfection, MKN-45 cells were collected and fixed in 70% ethanol at 4°C overnight. After washing, cells were incubated with 150 *μ*l RnaseA enzyme (final concentration 500 *μ*g/ml) and 150 *μ*l PI solution (final concentration 0.05 mg/ml) in the dark for 30 min and then analyzed by flow cytometry (FACS Calibur, BD Biosciences, CA, USA).

### 2.8. Apoptosis Analysis

Annexin V-FITC/PI Apoptosis Detection kit from Invitrogen (Carlsbad, CA, USA) was used. Briefly, at 48 h after transfection, MKN-45 cells were collected, resuspended in 1× binding buffer, and incubated with Annexin V-FITC for 15 minutes in dark at room temperature. The cells were then stained with PI dye solution for 30 minutes in dark. Cell apoptosis was measured by flow cytometry (FACS Calibur).

### 2.9. Statistical Analysis

PASW Statistics 18 software (SPSS, Inc., Chicago, IL, USA) was used in data analysis. Each experiment was repeated at least 3 times independently. The data were presented as mean ± SEM. The Student's *t*-test was used for comparison between the two groups. Multiple comparisons of samples were analyzed using one-way ANOVA and Post Hoc Multiple Comparisons (LSD). *P* < 0.05 was considered to have statistically significant difference.

## 3. Results

### 3.1. THAP11 Is Significantly Lower in GC Tissues and GC Cell Lines

**Figure 1:**
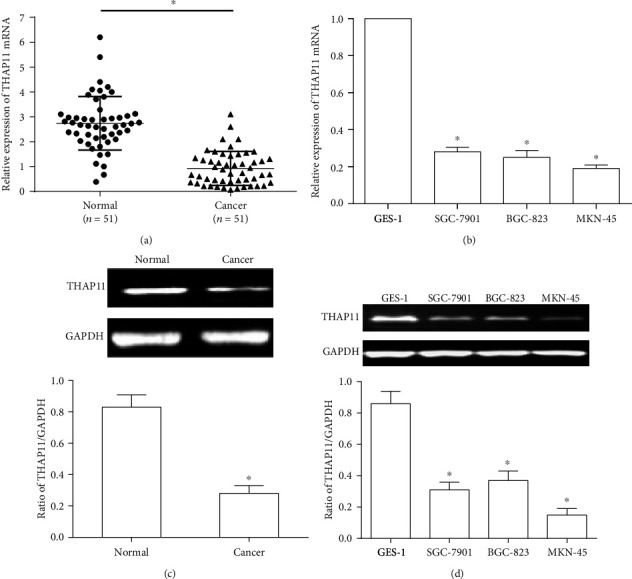
Analysis of THAP11 mRNA and protein expression in GC tissues and cell lines. (a) THAP11 mRNA expression in GC tissues was analyzed by qRT-PCR. (b) THAP11 mRNA expression in GC cell lines was analyzed by qRT-PCR. (c) THAP11 protein expression in GC tissues was analyzed by Western blot. GAPDH was used as an internal control. (d) THAP11 protein expression in GC cell lines was analyzed by Western blot. GAPDH was used as an internal control. Data are shown as mean ± SEM. Student's *t*-test or one-way ANOVA was used. Compared with normal or GES-1, ^∗^*P* < 0.05.

The THAP11 mRNA and protein expression in paired GC tissues and adjacent normal gastric tissues was detected with qRT-PCR and Western blot, respectively. As shown in Figures 1(a) and 1(c), THAP11 mRNA and protein expression were significantly lower in GC tissues than adjacent normal gastric tissues. To further verify the expression pattern of THAP11 in GC, we analyzed its expression in GC cell lines. Compared with GES-1, THAP11 mRNA and protein levels were decreased in all three GC cell lines (BGC-823, SGC-7901, and MKN-45) (Figures 1(b) and 1(d)). Therefore, expression is decreased in GC tissues and cell lines. In addition, the expression of THAP11 was lowest in MKN-45 cells. Thus, further analysis was performed in MKN-45 cells.

### 3.2. THAP11 Inhibits GC Cell Growth *In Vitro*

**Figure 2:**
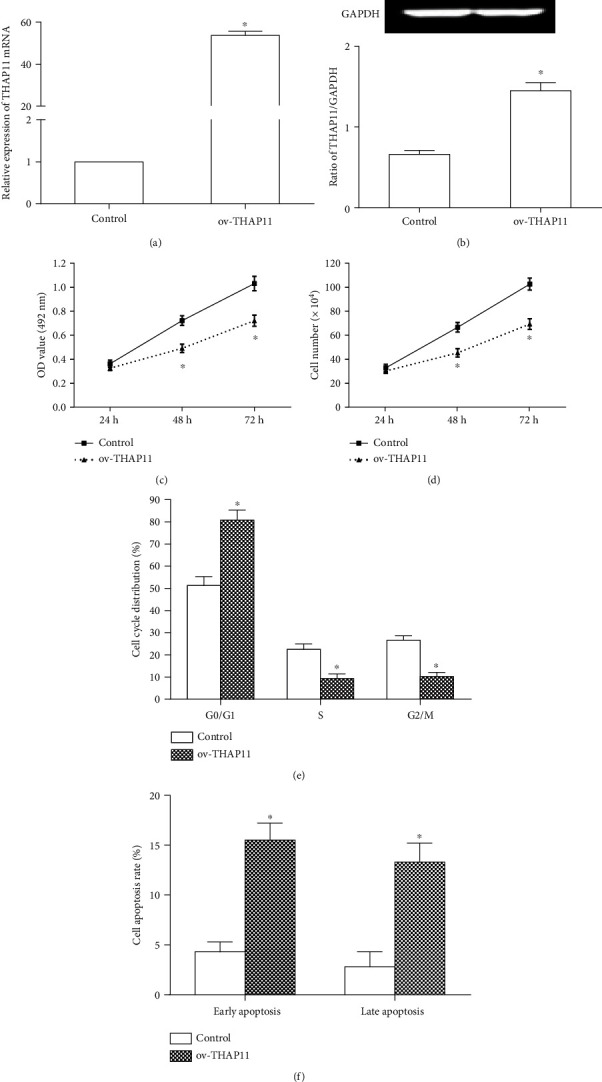
Effects of THAP11 overexpression on GC cell growth, cell cycle, and apoptosis *in vitro*. (a, b) MKN-45 cells were transfected with Ov-THAP11 or control vector. Expression of THAP11 was detected by qRT-PCR at 24 h after transfection and by Western blot at 48 h after transfection. GAPDH was used as an internal control. (c) MTT assay was used to detect the proliferation of MKN-45 cells at 24 h, 48 h, and 72 h after transfection, respectively. (d) After transfection, cell number was counted at 24 h, 48 h, and 72 h, respectively. (e) At 24 h after transfection, cell cycle was detected by flow cytometry. The graph shows the percentage of cells in each phase. (f) At 48 h after transfection, cell apoptosis was analyzed using the Annexin V-FITC apoptosis detection kit. The graph shows the early and late apoptosis. Data are shown as mean ± SEM. Compared with control, ^∗^*P* < 0.05, Student's *t*-test.

To investigate the effect of THAP11 on cell proliferation, cycle, and apoptosis of GC cells, MKN-45 cells were first transfected with THAP11 overexpressing plasmid (ov-THAP11). The transfection efficiency was similar between control and ov-THAP11 groups (data not shown). Figures 2(a) and 2(b) show that THAP11 was successfully overexpressed. Results of MTT assay and cell number counting showed that ov-THAP11 effectively inhibited the proliferation of GC cells (Figures 2(c) and 2(d)). Moreover, the representative flow cytometry results of cell cycle and apoptosis were shown in supplementary Figures 1A and 1B, respectively. We found that ov-THAP11 induced cell cycle arrest in G1/G0 and promoted apoptosis (Figures 2(e) and 2(f)).

**Figure 3:**
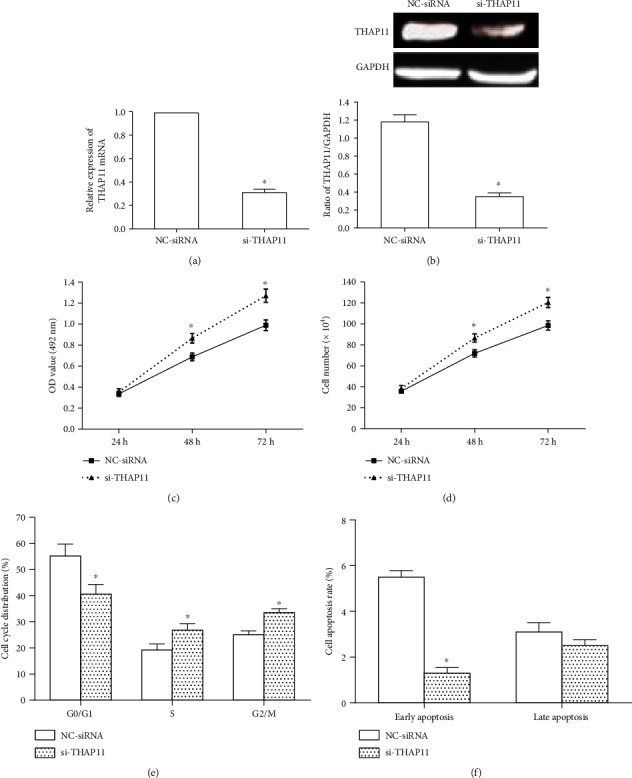
Effects of THAP11 silencing on GC cell growth, cell cycle, and apoptosis *in vitro*. (a, b) MKN-45 cells were transfected with si-THAP11 or control (NC-siRNA). Expression of THAP11 was detected by qRT-PCR at 24 h after transfection and by Western blot at 48 h after transfection. GAPDH was used as an internal control. (c) After transfection, MTT assay was used to detect the proliferation of MKN-45 cells at 24 h, 48 h and 72 h, respectively. (d) After transfection, cell number was counted at 24 h, 48 h, and 72 h, respectively. (e) At 24 h after transfection, cell cycle after was detected by flow cytometry. The graph shows the percentage of cells in each phase. (f) At 48 h after transfection, cell apoptosis was analyzed using the Annexin V-FITC apoptosis detection kit. The graph shows the early and late apoptosis of cells. Data are shown as mean ± SEM. Compared with NC-siRNA, ^∗^*P* < 0.05, Student's *t*-test.

Furthermore, RNA interference was conducted to silence endogenous THAP11 expression in MKN-45 cells. The transfection efficiency was similar between NC-siRNA and si-THAP11 groups (data not shown). Transfection of MKN-45 cells with si-THAP11 (40 nM) reduced THAP11 mRNA level (Figure 3(a)) and protein level (Figure 3(b)), indicating the effective knockdown of THAP11. Compared with the control group, si-THAP11 significantly promoted cell proliferation (Figures 3(c) and 3(d)) and increased DNA synthesis and cell division (Figure 3(e)), while inhibited apoptosis (Figure 3(f)). The representative flow cytometry results of cell cycle and apoptosis were shown in supplementary Figures 2A and 2B, respectively. Together, THAP11 suppresses MKN-45 cell growth.

### 3.3. THAP11 Suppresses c-Myc Expression and Regulates c-Myc Target Genes

**Figure 4:**
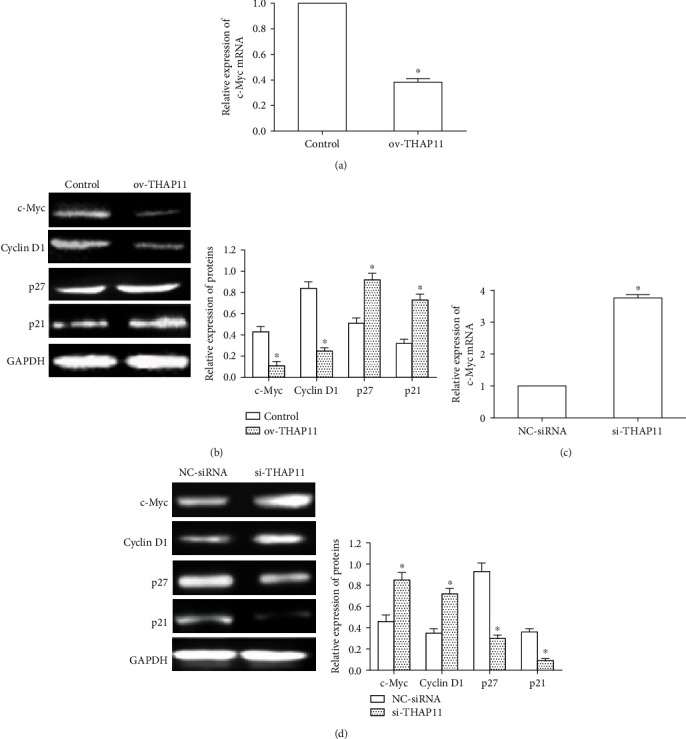
Effects of THAP11 overexpression on c-Myc expression and regulated c-Myc target genes. (a) MKN-45 cells were transfected with control or ov-THAP11, and c-Myc mRNA level was analyzed by qRT-PCR at 24 h after transfection. (b) Expression of c-Myc, cyclinD1, p27, and p21 protein was detected by Western blot at 48 h after transfection and the relative expression of proteins was quantitatively analyzed. (c) MKN-45 cells were transfected with NC-siRNA or si-THAP11, and c-Myc mRNA level was analyzed by qRT-PCR at 24 h after transfection. (d) Expression of c-Myc, cyclinD1, p27, and p21 protein was detected by Western blot at 48 h after transfection, and the relative expression of proteins was quantitatively analyzed. GAPDH was used as an internal control. Data are shown as mean ± SEM. Compared with control, ^∗^*P* < 0.05, Student's *t*-test.

Evidence indicates that the expression of c-Myc gene is closely related to cell proliferation and that the decrease in c-Myc expression inhibits cell growth [12, 13]. To investigate the relationship between THAP11 and c-Myc, we transfected the ov-THAP11 plasmids into MKN-45 cells and observed changes in c-Myc mRNA and protein levels. The transfection efficiency was similar between control and ov-THAP11 groups (data not shown). As shown in Figure 4(a), expression of c-Myc mRNA in the ov-THAP11 transfected group was decreased by 60% compared with thecontrol group. The c-Myc can regulate the expression of genes that are involved in metabolism, macromolecular synthesis, cell cycle, and apoptosis [14]. Among the target genes, cyclinD1, p27Kip1, and p21 are cell cycle regulators. Therefore, we examined the expression of c-Myc, cyclinD1, p27Kip1, and p21 proteins using Western blot. Ov-THAP11 transfection resulted in an obvious decrease in cyclinD1 protein but obvious increase in p27 and p21 proteins (Figure 4(b)). Moreover, we performed RNA interference to knockdown THAP11 expression and obtained similar results. Transfection of MKN-45 cells with si-THAP11 increased the levels of c-Myc mRNA (Figure 4(c)), c-Myc protein (Figure 4(d)), and cyclinD1 protein expression (Figure 4(d)) while decreased p27 and p21 protein expression (Figure 4(d)). Therefore, THAP11 can regulate the expression of genes involved in cell cycle regulation through modulating c-Myc expression.

### 3.4. THAP11-Induced Cell Growth Suppression Is Rescued by c-Myc Overexpression

**Figure 5:**
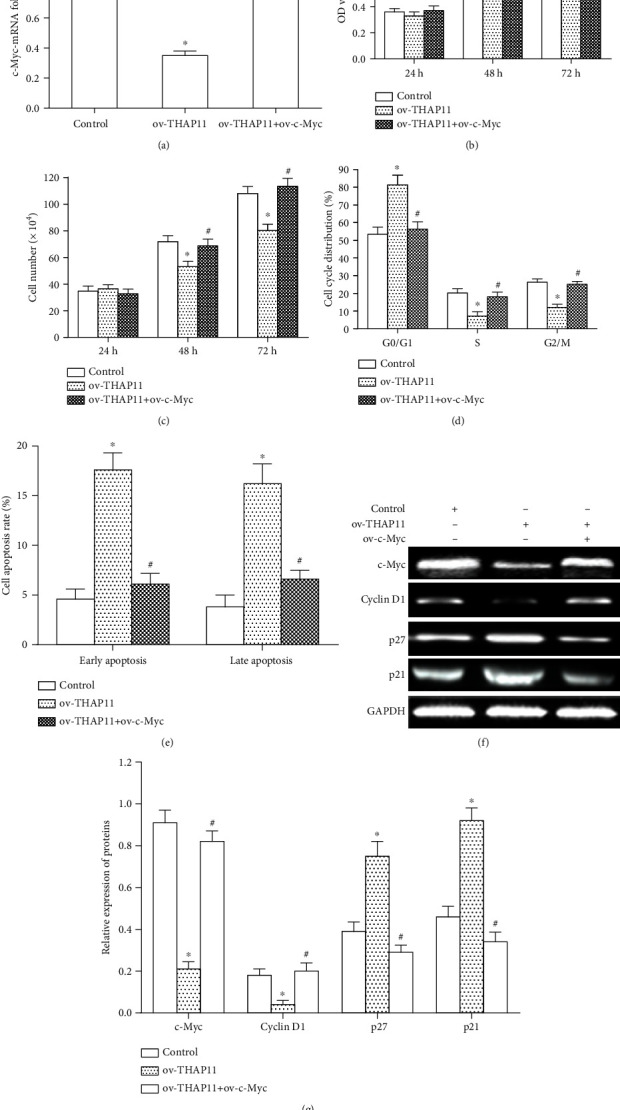
Overexpression of c-Myc rescued THAP11-induced cell growth inhibition. MKN-45 cells were transfected with both ov-THAP11 and ov-c-Myc, single ov-THAP11 or control. (a) c-Myc mRNA level was analyzed by qRT-PCR at 24 h after transfection. (b) MTT assay was used to detect the proliferation of cells at 24 h, 48 h, and 72 h after transfection, respectively. (c) Cell number was counted at 24 h, 48 h, and 72 h, respectively. (d) Cell cycle after was detected by flow cytometry at 24 h after transfection. The graph shows the percentage of cells in each phase. (e) Cell apoptosis was analyzed using the Annexin V-FITC apoptosis detection kit at 48 h after transfection. The graph shows the early and late apoptosis of cells. GAPDH was used as an internal control. (f) Expressions in c-Myc and its target genes in transfected cells were analyzed by Western blot at 48 h after transfection. (g) The bar graph shows the results of relative quantitative analysis of protein expression. GAPDH was used as an internal control. Data are shown as mean ± SEM. Compared with control, ^∗^*P* < 0.05; compared with ov-THAP11, ^#^*P* < 0.05, one-way ANOVA.

To examine whether THAP11 inhibits the proliferation of GC cell through c-Myc, we simultaneously transfected ov-THAP11 and ov-c-Myc into MKN-45 cells. The transfection efficiency was similar among control, ov-THAP11 and ov-c-Myc groups (data not shown). As shown in Figures 5(a)–5(d), cotransfection of ov-c-Myc with ov-THAP11 rescued the effects of ov-THAP11. In detail, compared with ov-THAP11 transfection alone, cotransfection of ov-c-Myc with ov-THAP11 significantly upregulation of c-Myc mRNA levels (Figure 5(a)), promoted cell proliferation (Figures 5(b) and 5(c)), and cell cycle progression (Figure 5(d)), while inhibited apoptosis (Figure 5(e)). The representative flow cytometry results of cell cycle and apoptosis were shown in supplementary Figures 3A and 3B, respectively. Using Western blot, we also confirmed that cotransfection of ov-c-Myc with ov-THAP11 increased cyclinD1 protein expression and decreased P27 and P21 protein expression (Figures 5(f) and 5(g)). Thus, THAP11 may inhibit GC cell growth by negative regulation of c-Myc expression.

## 4. Discussion

Some members of the human THAP protein family play a major role in cell cycle and proliferation and are closely related to the development of certain diseases. For example, THAP0, also known as death-related protein, can interact with p58ipk to phosphorylate-related proteins, blocking cell protein synthesis and inhibiting cell proliferation [15]. THAP1 can inhibit cell growth and cause G1 arrest by downregulating some pRB/E2 target genes [16]. THAP5 is a cell cycle inhibitor and closely related to certain heart diseases [17]. THAP2, 10, and 11 are involved in cancer development [18].

THAP11 plays an important role in cell growth [8, 12, 13]. However, its role and mechanism in the development of GC remains largely unknown. The present study investigated the effect of THAP11 on GC cell proliferation. We confirmed that THAP11 level was reduced in GC tissues and GC cells. The growth of GC cell line MKN-45 was significantly inhibited by THAP11 overexpression while promoted by THAP11 knock down. A study has shown that proliferation of HepG2 hepatic carcinoma cells significantly increased after endogenous THAP11 knockout [12]. This is consistent with our results. However, in SW620 colon cancer cells, THAP11 knockdown led to a marked decrease in cell proliferation [8]. These results confirm the important role of THAP11 in cell growth.

c-Myc is a protooncogene that plays a nonnegligible role in cell cycle regulation, apoptosis, and tumorigenesis [14, 19]. Studies have reported that transcription factors such as hPLSCR1, E2F4, RBM38, BRD4, and hZ*α*ADAR1 can regulate c-Myc expression via directly binding to its promoter region [20–24]. Several key target genes of c-Myc have been identified, including cyclins and cyclin-dependent kinases (Cdks) [25]. As a transcription factor regulating c-Myc expression, THAP11 directly binds to specific sites in the c-Myc promoter region to inhibit the transcription of c-Myc and further downregulates c-Myc downstream target genes such as cyclinD1 while upregulates the cyclin-dependent kinase inhibitor p21^Cip1^, further inhibiting cell growth [12]. Recently, the importance of the THAP11/c-Myc pathway in the proliferation of chronic myogenic leukemia progenitor cells has been elucidated [13]. Similarly, the function of this pathway has been confirmed in human breast cancer [12], liver cancer [7], and colon cancer [8] cells. Our current data showed that THAP11 decreased c-Myc protein expression, which in turn reduced cyclin D1 expression and increased p27 and p21 protein levels. Various studies have shown that one of the reasons for the loss of cell cycle control and the occurrence of malignant tumors is related to the abnormal overexpression of the cyclinD1 [26, 27]. The p27Kip1 gene is a novel target of c-Myc in regulating cell proliferation and tumor transformation. c-Myc inhibits the CDK inhibitor p27 by directly inhibiting its gene transcription or indirectly promoting ubiquitin-mediated degradation [28]. p21 is a main target of c-Myc [25], and the downregulation of p21 expression is closely related to the recovery of cell proliferation control [29]. However, it should be noted that expression of p21 and p27 is also regulated by a c-Myc-independent mechanism [30]. Taken together, downregulation of c-Myc and its modulation of target gene (cyclin D1, p27, and p21) expression may be a key mechanism for THAP11-mediated growth arrest in GC cells.

## 5. Conclusions

In summary, THAP11 plays an important role as tumor suppressor in the pathogenesis of GC, and it negatively regulates c-Myc oncogene expression possibly through transcriptional repression. Therefore, the THAP11/c-Myc pathway may be an indispensable pathway in regulating GC cell proliferation. However, more experiment evidence is needed to further verify the role of THAP11/c-Myc pathway in regulating GC cell proliferation.

## Figures and Tables

**Table 1:** Sequences of siRNA.

Name	Sequence
Negative siRNA (NC-siRNA) sense	5′-UUCUCCGAACGUGUCACGU TT-3′
Negative siRNA (NC-siRNA) antisense	5′-ACGUGACACGUUCGGAGAATT-3′
THAP11 siRNA sense	5′-GCUGCACUUCUACACGUUUTT-3′
THAP11 siRNA antisense	5′-AAACGUGUAGAAGUGCAGCTT-3′

**Table 2:** Primer sequences used for qRT-PCR.

Gene	Sequence
THAP11-forward	5′-ATGCGCAAAGTAGCGCGCAGACCC-3′
THAP11-reverse	5′-CGGGCAGAGGGTGAGGACTGCTG-3′
c-Myc-forward	5′-TGAGGAGACACCGCCCAC-3′
c-Myc-reverse	5′-CAACATCGATTTCTTCCTCATCTTC-3′
GAPDH-forward	5′-GAAGGTGAAGGTCGGAGTCA-3′
GAPDH-reverse	5′-TTGAGGTCAATGAAGGGGTC-3′

**Table 3:** Information on antibodies used for Western blot.

Antibody (catalog)	Dilution	Specificity	Company
THAP11 (23030-1-AP)	1 : 1000	Rabbit polyclonal	Proteintech Group
c-Myc (10828-1-AP)	1 : 1000	Rabbit monoclonal	Proteintech Group
Cyclin D1 (60186-1-lg)	1 : 1000	Mouse monoclonal	Proteintech Group
p27 (25614-1-AP)	1 : 1000	Rabbit monoclonal	Proteintech Group
p21 (10355-1-AP)	1 : 1000	Rabbit monoclonal	Proteintech Group
GAPDH (10494-1-AP)	1 : 1000	Rabbit monoclonal	Proteintech Group
IgG (111-035-144)	1 : 5000	Rabbit polyclonal	Jackson ImmunoResearch Laboratories, Inc.

## Data Availability

The data used to support the findings of this study is available upon request.

## Supplementary Materials

Supplementary Materials Supplementary Figure 1: representative flow cytometry results. (a) Representative flow cytometry results of cell cycle in each group. (b) Representative flow cytometry results of apoptosis in each group. Supplementary Figure 2: representative flow cytometry results. (a) Representative flow cytometry results of cell cycle in each group. (b) Representative flow cytometry results of apoptosis in each group. Supplementary Figure 3: representative flow cytometry results. (a) Representative flow cytometry results of cell cycle in each group. (b) Representative flow cytometry results of apoptosis in each group.
