# Supplementary material for: THAP11 Functions as a Tumor Suppressor in Gastric Cancer through Regulating c-Myc Signaling Pathways
Source: Biomed Res Int. 2020 Aug 27;2020:7838924. doi: 10.1155/2020/7838924 (PMC7474744; doi:10.1155/2020/7838924)
Supplement: Supplementary Materials — Supplementary Figure 1: representative flow cytometry results. (a) Representative flow cytometry results of cell cycle in each group. (b) Representative flow cytometry results of apoptosis in each group. Supplementary Figure 2: representative flow cytometry results. (a) Representative flow cytometry results of cell cycle in each group. (b) Representative flow cytometry results of apoptosis in each group. Supplementary Figure 3: representative flow cytometry results. (a) Representative flow cytometry results of cell cycle in each group. (b) Representative flow cytometry results of apoptosis in each group. [file 7838924.f1.pdf]

Supplementary Fig. 1

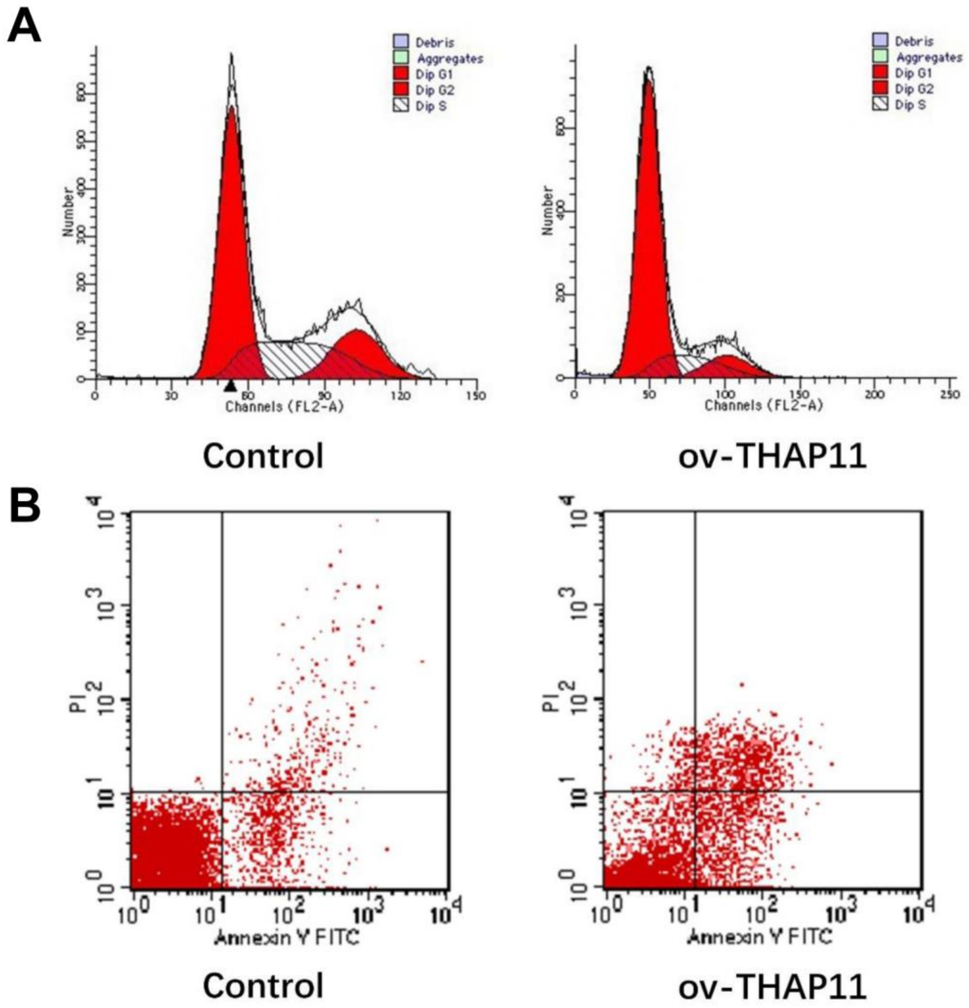

Supplementary Fig. 1. Representative flow cytometry results.

(A) Representative flow cytometry results of cell cycle in each group. (B) Representative flow cytometry results of apoptosis in each group.

Supplementary Fig. 2

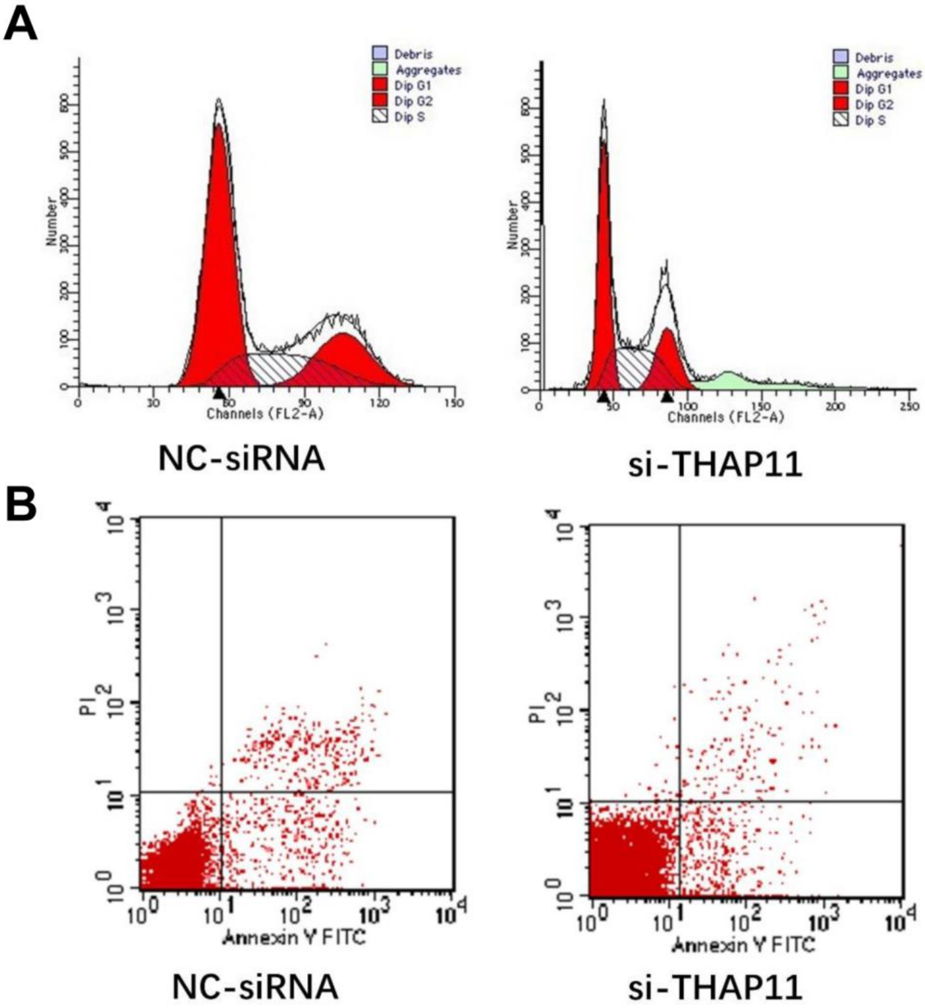

Supplementary Fig. 2. Representative flow cytometry results.
(A) Representative flow cytometry results of cell cycle in each group. (B) Representative flow cytometry results of apoptosis in each group.

Supplementary Fig. 3

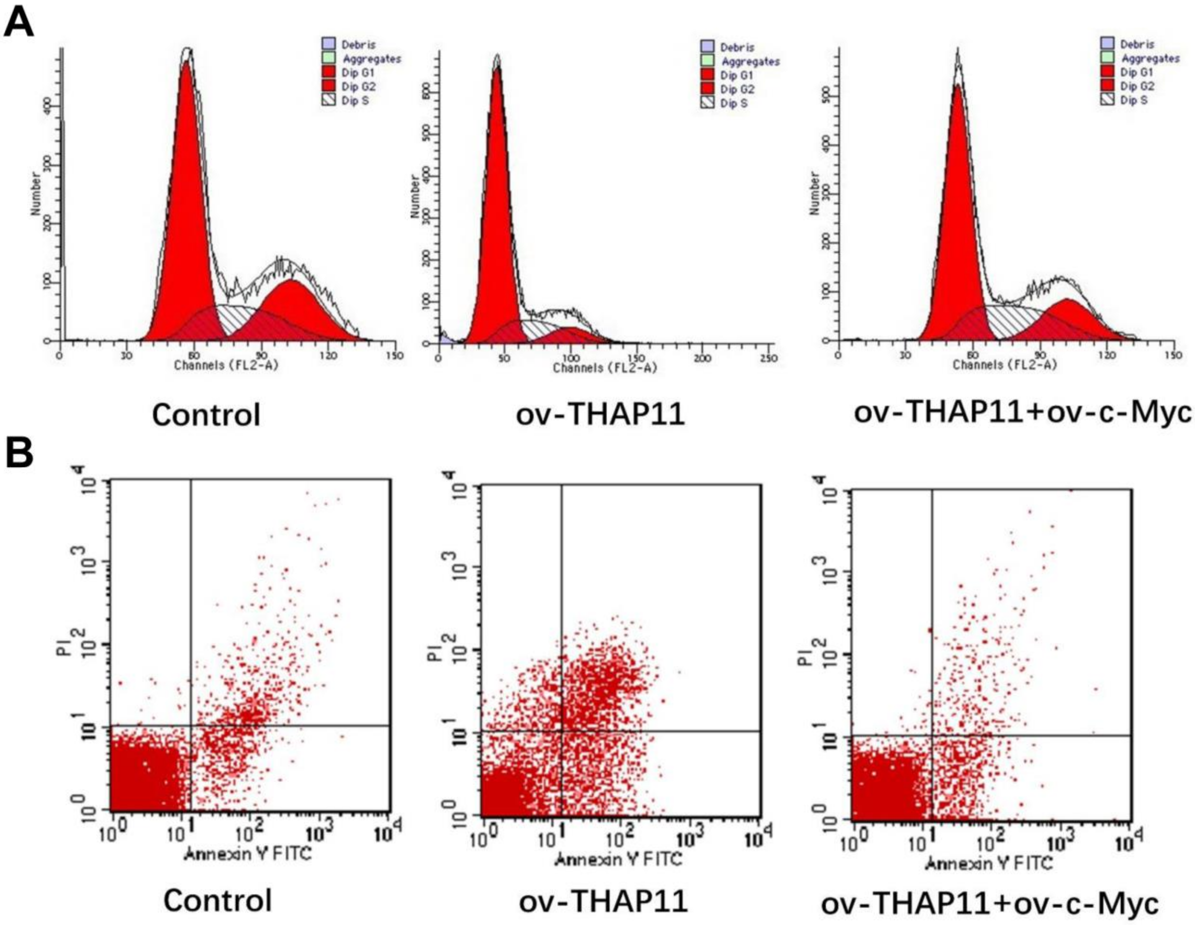

Supplementary Fig. 3. Representative flow cytometry results.

(A) Representative flow cytometry results of cell cycle in each group. (B) Representative flow cytometry results of apoptosis in each group.
